# Antisense oligonucleotides extend survival of prion-infected mice

**DOI:** 10.1172/jci.insight.131175

**Published:** 2019-08-22

**Authors:** Gregory J. Raymond, Hien Tran Zhao, Brent Race, Lynne D. Raymond, Katie Williams, Eric E. Swayze, Samantha Graffam, Jason Le, Tyler Caron, Jacquelyn Stathopoulos, Rhonda O’Keefe, Lori L. Lubke, Andrew G. Reidenbach, Allison Kraus, Stuart L. Schreiber, Curt Mazur, Deborah E. Cabin, Jeffrey B. Carroll, Eric Vallabh Minikel, Holly Kordasiewicz, Byron Caughey, Sonia M. Vallabh

**Affiliations:** 1 Laboratory of Persistent Viral Diseases, Rocky Mountain Laboratories, National Institute of Allergy and Infectious Diseases, NIH, Hamilton, Montana, USA.; 2 Ionis Pharmaceuticals Inc., Carlsbad, California, USA.; 3 Broad Institute of MIT and Harvard, Cambridge, Massachusetts, USA.; 4 McLaughlin Research Institute, Great Falls, Montana, USA.; 5 Western Washington University, Bellingham, Washington, USA.; 6 Program in Biological and Biomedical Sciences, Harvard Medical School, Boston, Massachusetts, USA.; 7 Prion Alliance, Cambridge, Massachusetts, USA.

**Keywords:** Neuroscience, Therapeutics, Gene therapy, Neurodegeneration, Prions

## Abstract

Prion disease is a fatal, incurable neurodegenerative disease of humans and other mammals caused by conversion of cellular prion protein (PrP^C^) into a self-propagating neurotoxic conformer (prions; PrP^Sc^). Strong genetic proofs of concept support lowering PrP expression as a therapeutic strategy. Antisense oligonucleotides (ASOs) can provide a practical route to lowering 1 target mRNA in the brain, but their development for prion disease has been hindered by 3 unresolved issues from prior work: uncertainty about mechanism of action, unclear potential for efficacy against established prion infection, and poor tolerability of drug delivery by osmotic pumps. Here, we test ASOs delivered by bolus intracerebroventricular injection to intracerebrally prion-infected WT mice. Prophylactic treatments given every 2–3 months extended survival times 61%–98%, and a single injection at 120 days after infection, near the onset of clinical signs, extended survival 55% (87 days). In contrast, a nontargeting control ASO was ineffective. Thus, PrP lowering is the mechanism of action of ASOs effective against prion disease in vivo, and infrequent — or even single — bolus injections of ASOs can slow prion neuropathogenesis and markedly extend survival, even when initiated near clinical signs. These findings should empower development of PrP-lowering therapy for prion disease.

## Abstract 2

ASO-mediated prion protein suppression delays disease and extends survival, even in mice with established prion infection.

## Graphical abstract

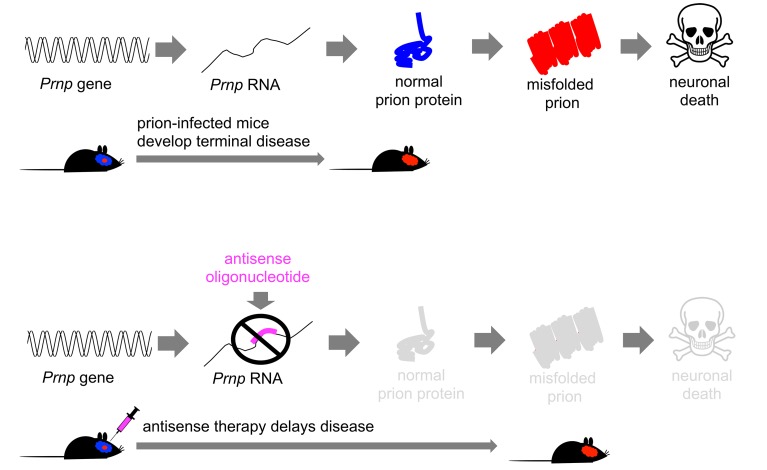

## Introduction

Human prion disease is an incurable, uniformly fatal neurodegenerative disease that typically presents as a rapidly progressive dementia. Regardless of etiology — sporadic, genetic, or acquired — and regardless of clinical name — Creutzfeldt-Jakob disease, fatal familial insomnia, or Gerstmann-Sträussler-Scheinker disease — all prion disease shares a single molecular mechanism. All cases arise from conformational change of the prion protein (PrP), encoded in humans by the gene *PRNP* (1), from its native fold (cellular prion protein; PrP^C^) to a self-propagating misfolded form (scrapie prion protein; PrP^Sc^). Lowering PrP levels should be an effective treatment for prion disease because PrP KO and postnatal suppression are known to confer protection from prion disease (2–4), and *Prnp* gene dosage is correlated with the pace of disease across a wide range of models and expression levels (5–7). PrP lowering should also be well tolerated, as PrP-KO mice, goats, and cattle are viable, fertile, have normal lifespans, and exhibit normal behavior (8–10), and humans with heterozygous loss-of-function variants in *PRNP* are healthy (11, 12). The 1 phenotype that appears reliably attributable to PrP KO, a mild sensorimotor defect caused by lack of stimulation of myelin maintenance in peripheral nerves by a PrP-derived signaling peptide, is not seen in heterozygous animals, nor in the CNS (13, 14).

Given the extensive genetic validation of this therapeutic hypothesis, there have been several efforts to develop PrP-lowering therapies, but RNA interference approaches have so far been limited by restricted brain uptake or distribution (15–18), and small molecules have not advanced beyond the initial discovery stage (19). Antisense oligonucleotides (ASOs) are 1 potential modality for reducing levels of a single target protein in the brain and are being advanced preclinically and clinically for several other neurodegenerative diseases (20–29). These short (17–20 base) single-stranded oligonucleotides, chemically modified for pharmacokinetic stability, are capable of impacting disease biology through specific modulation of complementary target RNAs (30, 31), including RNAse H–dependent degradation (32–34).

A previous study of 1 PrP-targeting ASO found some evidence of efficacy, with mice living 40% longer if treated the day after prion infection (35), but further development of ASOs for prion disease stalled due to 3 unresolved issues. First, the mechanism of action was unknown. While PrP RNA lowering was observed, consistent with the intended activity of ASOs (31), sequence-independent effects had also been observed on prion load in cell culture and even on prion infectivity in animals under certain conditions — for example, if prions were preincubated with phosphorothioate (PS) oligos prior to injection or injected into the periphery and then chased with PS oligo prior to neuroinvasion (35–37), consistent with PrP’s known affinity for polyanions (38, 39) and the known protein-binding properties of ASOs (40). This left ambiguity as to whether aptameric effects, rather than RNA lowering, drove the observed survival extension and whether a pharmacodynamic biomarker (41) could provide a meaningful metric of target engagement to support clinical development. Second, while efficacy was observed with treatment 1 day after prion infection, efficacy at a later time point could not be evaluated due to toxicity issues. This left unclear the potential for treatment of established prion infection, an important issue because many candidate antiprion compounds effective in early treatment have shown diminished or no efficacy later in disease (42–45). Third, half of mice were lost due to complications from delivery of ASOs by continuous infusion via osmotic pumps, and the path to translation of continuous infusion into a realistic human dosing regimen was not clear (35).

Since that time, ASOs have undergone additional deep characterization in the CNS of nonhuman primates (NHPs) and humans in the context of ASO drugs currently approved or in trials for spinal muscular atrophy, Huntington’s disease, amyotrophic lateral sclerosis, and tauopathies, among other neurological indications (22, 23, 46–49). This work has resulted in key advances in delivery of ASOs to the CNS, including the observation that periodic bolus dosing, achieved by stereotactic intracerebroventricular (i.c.v.) injection in rodents or by intrathecal delivery in NHPs and humans, results in better-tolerated administration and more sustained activity than continuous infusion (50). New data on ASO brain distribution, stability, and tolerability in NHP and human brain following bolus dosing (22, 46–48) support the relevance of this modality to a whole brain disease such as prion disease.

In light of these advances, we set out to revisit ASOs as a therapeutic approach for prion disease. In the present study, we demonstrate efficacy of ASOs following bolus dosing into prion-infected mice. We demonstrate that lowering of PrP RNA, and thereby PrP, is the mechanism of action by which ASOs confer efficacy against prion disease in vivo. We further show that ASO-mediated lowering of PrP confers therapeutic benefit, both prophylactically and after prion neuropathology, is well established in the brain, near the onset of frank clinical signs.

## Results

### Discovery and characterization of candidate ASOs.

**Figure 1:**
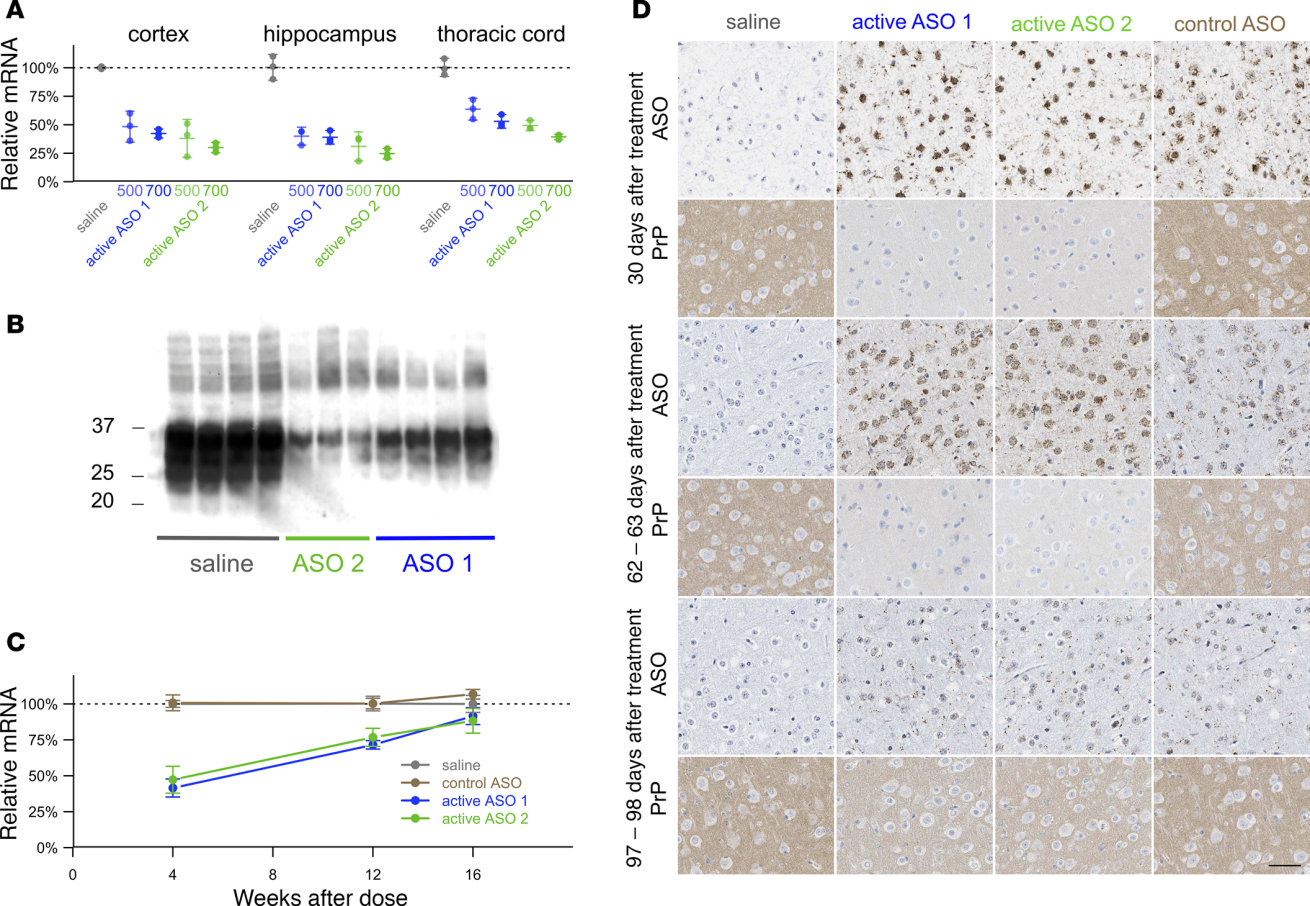
Potency and persistence of anti-PrP ASOs in the mouse brain. (**A**) ASOs were administered i.c.v. at 500 or 700 μg to groups of *n* = 3 mice, and ipsilateral hemispheres were collected 8 weeks later and microdissected for regional PrP RNA quantification by RT-PCR (Supplemental Table 1). Data were normalized to the mean value for saline-treated animals. Error bars indicate 95%CI of the mean. (**B**) Western blots from whole contralateral hemispheres of SWR/R mice 8 weeks after treatment with 700 μg ASO. (**C**) RT-PCR quantification of PrP RNA in the ipsilateral cortex of *n* = 4 mice at 4, 12, and 16 weeks after a single 500 μg ASO dose. Data were normalized to the mean value for saline-treated animals. Error bars indicate 95%CI of the mean. (**D**) IHC images of sagittal midline cortical sections stained using antibodies to PrP (D13) or the ASO backbone at the indicated number of days after a single 300 μg i.c.v. injection of ASO. Representative images of a total of 3 mice per treatment group per time point, except saline 62–63 days, for which 5 mice were analyzed. Scale bar: 50 μm.

We designed and screened ASOs complementary to mouse *Prnp* mRNA and identified 2 potent ASOs, termed active ASO 1 and active ASO 2, which respectively target the 3′ UTR and intron 2 of the mouse *Prnp* gene (Table 1 and Supplemental Figure 1A; supplemental material available online with this article; https://doi.org/10.1172/jci.insight.131175DS1). Stereotactic i.c.v. injection of 300 μg of either ASO resulted in reduction in *Prnp* mRNA in WT mice with tissue collection at 2 weeks after dosing in both the cortex and spinal cord (Supplemental Figure 1B). The ASOs were well-tolerated in naive WT C57BL/6N mice following a single i.c.v. bolus injection at 700 μg, with no significant difference from saline-treated animals in terms of body weight change or neuroinflammatory markers at 8 weeks after dose (Supplemental Figure 1, C and D). We further confirmed ASO activity in SWR/R mice following i.c.v. injection of ASOs at 500 μg or 700 μg, with collection of different brain regions at 8 weeks after dose for mRNA analysis. Substantial *Prnp* mRNA reduction was observed in the ipsilateral entorhinal cortex, hippocampus, and thoracic spinal cord in PrP ASO-treated mice compared with saline-treated mice (Figure 1A).

As expected, ASO-mediated reduction in PrP RNA was predictive of reduction in PrP protein levels, as measured by Western blot after i.c.v. ASO injection (Figure 1B and Supplemental Figure 2A). We also experimented with freehand intracerebral (i.c.) injections of ASO, and while this method did not provide dosing sufficiently consistent for survival studies, it revealed an inverse spatial relationship between ASO distribution and PrP distribution for ASOs targeting the PrP mRNA and not for the control ASO (Supplemental Figure 2, B and C). All of the above findings are consistent with RNAse H–mediated PrP mRNA lowering leading to decreased PrP synthesis and, thus, reduction in total PrP protein.

To enable survival studies in prion-infected mice, we also examined the duration of ASO activity in the mouse brain in order to determine an appropriate dosing regimen. After administration of a 500 μg bolus dose of active ASOs 1 or 2, cortical *Prnp* mRNA levels remained depressed through 84 days but largely rebounded by 112 days (Figure 1C). Bolus doses of 300 μg elicited similar results, with robust ASO staining and PrP^C^ reduction at 30 and 62 days after treatment, diminishing by 97–98 days (Figure 1D). For survival studies, we settled on dosing regimens of either 300 μg every 60 days (NIH) or 500 μg every 90 days (Broad Institute).

### Benefit of prophylactic ASO treatment against prion disease in mice.

We next sought to determine the efficacy of ASOs in a standard mouse model of prion disease: WT animals i.c. infected with the RML strain of prions. In the first set of experiments, we pursued a prophylactic dosing regimen, with the first ASO treatments at 14 days prior to infection.

**Figure 2:**
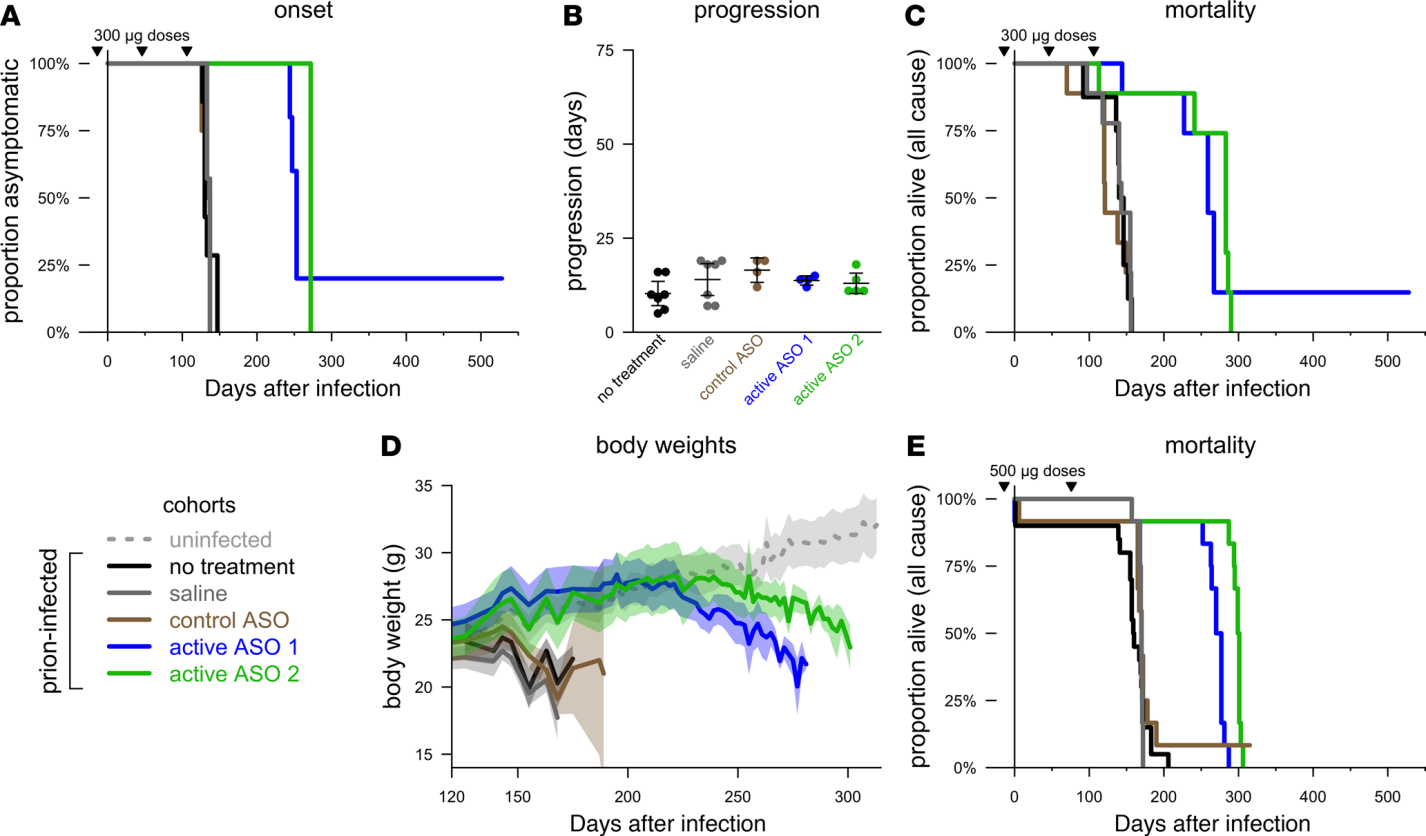
Benefit of prophylactic ASO treatment in prion-infected mice. (**A**) Delay of onset of clinical signs characteristic of prion disease in animals treated at NIH. Arrows indicate timing of 300 μg i.c.v. doses. One ASO 1–treated mouse at NIH showed no clinical signs prior to euthanasia at 527 dpi (Figure 2C); its brain was negative for prion pathology by IHC and for prion seeding activity by RT-QuIC (75), suggesting that either the infection was cleared or the original inoculum had failed to deliver an infectious dose. (**B**) Disease duration (onset to end stage) in animals treated at NIH. Bars indicate mean and 95%CI of the mean. (**C**) All-cause mortality in animals treated at NIH. (**D**) Body weights of animals treated at the Broad Institute. Lines indicate means, and shaded areas indicate 95%CI of the means. Only time points with ≥ 2 animals are included. (**E**) All-cause mortality in animals treated at the Broad Institute. One control ASO-treated mouse at Broad Institute showed no symptoms prior to euthanasia at 315 dpi; its brain was positive by RT-QuIC, with endpoint at a 1 × 10^–7^ dilution, suggesting it had eventually reached a prion titer similar to or slightly below that in terminal mice, but its survival > 15 SDs longer than the mean endpoint for its cohort suggests that the original inoculation had delivered an incomplete dose of prion infectivity. Arrows indicate timing of 500 μg i.c.v. doses. Treatment groups were *n* = 9 animals at NIH and *n* = 12 animals at Broad Institute, with some animals censored; see Supplemental Table 2 for details.

In experiments conducted at NIH, WT SWR/R mice received no treatment or i.c.v. injections of saline or 300 μg of control ASO, active ASO 1, or active ASO 2 at 14 days before infection and twice more at roughly 60-day intervals. Active ASOs 1 and 2 delayed the onset of prion disease clinical signs by 82% and 99%, respectively, compared with saline (median 250 and 272 days after infection [dpi] , respectively, vs. 137 dpi), whereas the control ASO provided no benefit, with onset and survival similar to saline-treated controls (Figure 2A). ASO-treated animals eventually succumbed to terminal prion disease and followed a standard clinical course, with the duration from first-observed clinical signs to terminal endpoint appearing similar to that of controls (Figure 2B). Four of 8 animals treated with control ASO died suddenly 8–9 days following the third dose. Several animals across other cohorts died intercurrently at various times for reasons apparently unrelated to prion disease, and some animals were also sacrificed for comparative histology; a complete accounting of animal numbers is included in Supplemental Table 2. Despite the intercurrent deaths, active ASOs 1 and 2 delayed all-cause mortality by 81% and 98%, respectively, compared with saline (median 259 and 283 dpi, respectively, vs. 143 dpi; Figure 2C), confirming the clinical benefit of PrP-lowering ASOs.

In experiments conducted at the Broad Institute, WT C57BL/6N mice received no treatment or i.c.v. injections of saline or 500 μg of control ASO, active ASO 1, or active ASO 2 at 14 days before infection and again 76 dpi. Active ASOs 1 and 2 delayed body weight loss (Figure 2D) and extended survival (all-cause) by 61% and 76%, respectively, compared with saline (median 274 and 300 dpi, respectively, vs. 170 dpi), with control ASO providing no benefit (Figure 2E). These experiments required only 2 surgeries, and the only intercurrent deaths observed were immediately following prion inoculation (3 mice total across all treatment cohorts). Strikingly, the survival benefit of active ASOs 1 and 2 were closely replicated in these 2 alternative study designs, as was the lack of benefit conveyed by the control ASO. Together, these data indicate that ASO-mediated PrP suppression delays prion disease onset and extends life when administered prophylactically.

### Benefit of ASO treatment against established prion disease in mice.

**Figure 3:**
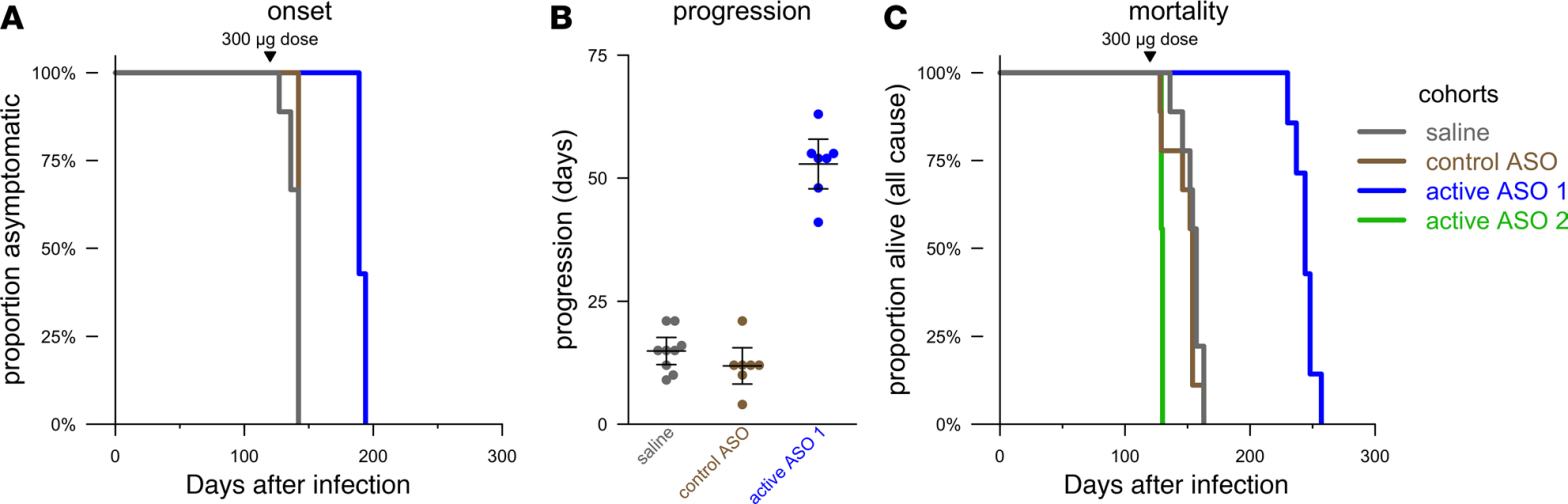
Benefit of ASO treatment against established prion infection in mice. (**A**) Delay of clinical onset in animals treated at NIH. Arrow indicates timing of single 300 μg i.c.v. dose. (**B**) Disease duration (onset to end stage) in animals treated at NIH. Bars indicate mean and 95%CI of the mean. (**C**) All-cause mortality in animals treated at NIH. Arrow indicates timing of single 300 μg i.c.v. dose. Treatment groups were *n* = 9 animals.

While the above experiments suggested the potential for efficacy in prophylactic intervention in prion disease, we also sought to determine whether ASOs could be effective against established prion infection. Moreover, any therapeutically relevant aptameric interaction between PrP^Sc^ and ASOs might only be observable at a late time point when PrP^Sc^ has accumulated in the brain, making later treatment experiments essential for interrogating mechanism of action. We therefore treated WT SWR/R mice at 120 dpi, a time point when prion neuropathology is already prominent and approximately 15 days before the expected onset of frank clinical signs. At this stage of advanced pathology, active ASO 2 was not tolerated, and all animals experienced sudden decline resulting in death or euthanasia (within roughly 16 hours) 8–9 days after surgery. This was also observed in some (2 of 9 mice) control ASO mice. The remainder of control ASO mice succumbed to disease at the same time as saline-treated controls, confirming PrP lowering as the relevant mechanism of action. Active ASO 1 delayed the onset of prion disease clinical signs by 33% (median 189 vs. 142 dpi; Figure 3A) and resulted in slower progression of symptomatic disease, with a clinical phase more than 3 times longer than in saline-treated mice (onset to end stage 53 ± 7 days vs. 15 ± 4 days, mean ± SD; Figure 3B). Overall, active ASO 1 at 120 dpi increased survival time in terms of all-cause mortality by 55% compared with saline (median 244 dpi vs. 157 dpi; Figure 3C). These data indicate that PrP-lowering ASOs can be identified that are both tolerated at a pathological time point and capable of extending survival when administered at this stage by delaying onset of clinical signs as well as by slowing symptomatic progression.

### Effects of ASO treatment on prion disease neuropathology.

**Figure 4:**
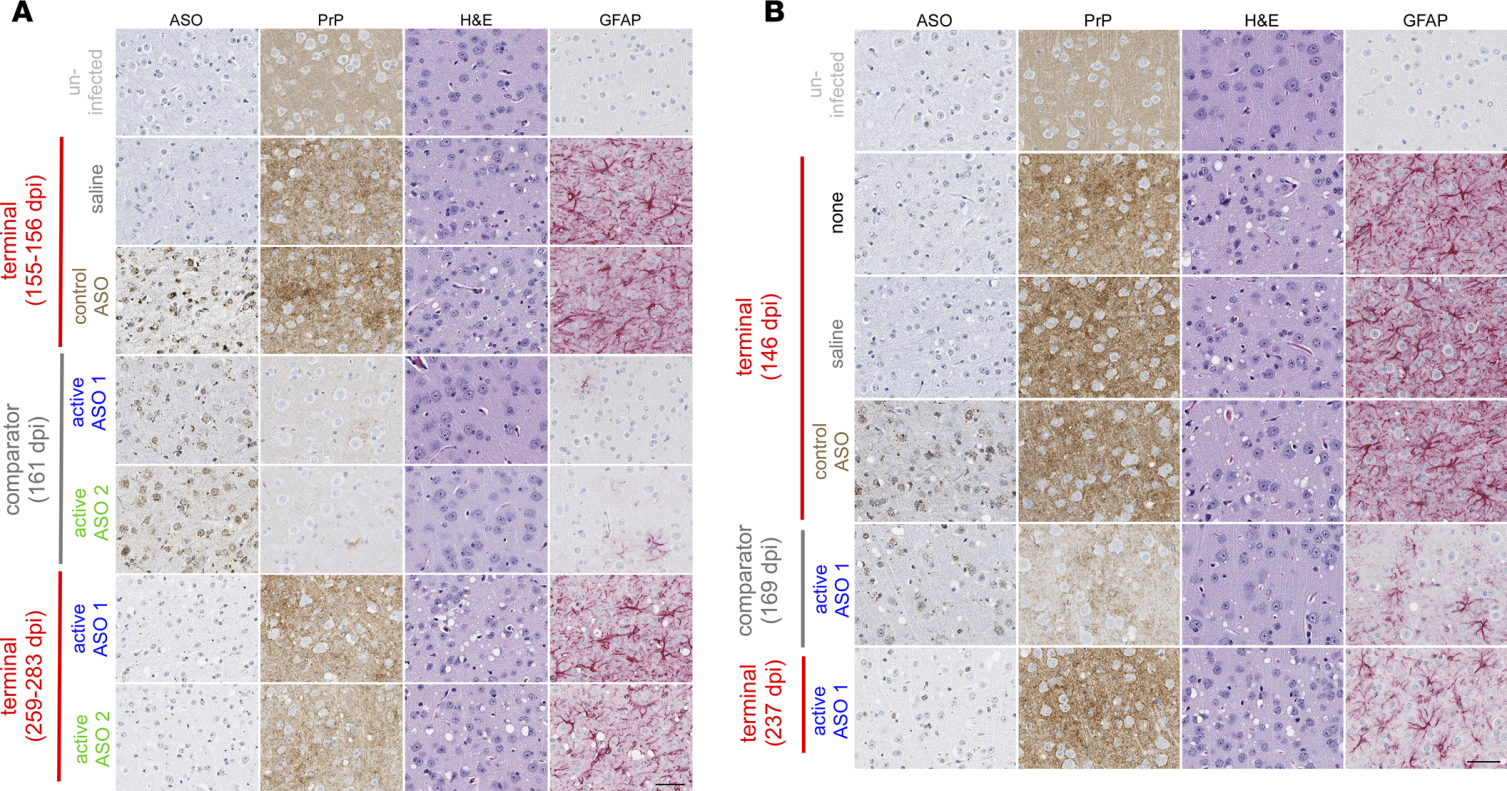
Effects of PrP-lowering ASOs on prion neuropathology. (**A** and **B**) Distributions of ASO, PrP, spongiosis (H&E), and astrogliosis (GFAP) in representative mice treated prophylactically (**A**) and at 120 dpi (**B**). Scale bar: 50 μm. Images are representative of a total of (**A**) 25 mice, including 7 saline, 4 control ASO, 5 each active ASO 1 and 2 terminal, and 2 each active ASO 1 and 2 comparator; and (**B**) 28 mice, including 3 no treatment, 9 saline, 7 control ASO, 7 active ASO 1 terminal, and 2 active ASO 1 comparator. Pixel counts from additional mice are provided in Supplemental Figures 3 and 5, and Western blots of PK-resistant PrP are provided in Supplemental Figures 4 and 6.

Brains of treated and control animals in both the prophylactic and 120 dpi experiments at NIH were analyzed for prion disease pathological changes by histology (Figure 4, Supplemental Figure 3, and Supplemental Figure 5) and immunoblotting for proteinase K–resistant (PK-resistant) PrP (Supplemental Figure 4 and Supplemental Figure 6). As compared with uninfected controls, terminal control mice (both saline and control ASO–treated) showed pathological changes consistent with advanced prion disease, such as granular PrP deposits, spongiform vacuolation, astrogliosis, and elevated PK-resistant PrP levels (Figure 4). Brains collected contemporaneously from still-asymptomatic mice treated prophylactically with ASOs 1 and 2 or at 120 dpi with ASO 1 showed the presence of ASO and reduced levels of all pathological changes, including PrP deposits (Figure 4, A and B). When treated animals eventually advanced to terminal prion disease, their neuropathology resembled that of untreated animals at endpoint (Figure 4, A and B, and Supplemental Figures 3–6), with similar levels of spongiform change and PrP deposition, albeit slightly attenuated astrogliosis. Together, these results indicate that PrP-lowering ASOs delay prion disease by slowing the accumulation of misfolded PrP and attendant neuropathological changes. The control ASO had no discernible effects on neuropathology at either time point.

## Discussion

Here, we demonstrate the efficacy of PrP-lowering ASOs against prion disease in mice, addressing 3 questions that hindered the development of ASOs for prion disease. First, we find that efficacy against prion disease is achieved by 2 PrP-targeting ASOs, but not by a control ASO, demonstrating that ASO efficacy is due to lowering of PrP RNA and not due to aptameric interaction (35–37) between ASOs and PrP. This distinction is important because the PrP-lowering mechanism lends itself to measurement of PrP in cerebrospinal fluid (CSF) as a pharmacodynamic biomarker (41, 51). Second, we show a substantial survival benefit in mice treated with a single dose of a PrP-lowering ASO 120 dpi, when neuropathology is prominent and clinical signs imminent. This supports the relevance of ASO treatment at a time point of established prion infection, extending earlier findings in conditional expression models (3, 4). Third, we find that bolus i.c.v. dosing is effective. This route of delivery into the CSF in rodents achieves similar brain distribution as intrathecal dosing in primates (50), suggesting a potential for periodic human dosing by lumbar puncture, similar to the regimen being used clinically for other ASOs (29).

The previous study of ASOs in prion disease reported sudden deaths in mice treated 60 days after prion infection (35). Our data recapitulate this phenomenon, as we observed sudden decline requiring euthanasia within 2 weeks after i.c.v. injection of active ASO 2 at 120 dpi. Our data show that this is not an on-target effect of PrP lowering, both because active ASO 1 was tolerated at 120 dpi and because the control ASO, which does not lower PrP, elicited a similar response in some animals (sudden decline in 4 of 8 mice after the third dose in the prophylactic study and 2 of 9 mice in the 120 dpi study). The ASOs used in these experiments were not human clinical candidates but were rather proof-of-concept ASOs against the mouse *Prnp* gene that had passed only minimal tolerability screening. The underlying prion pathology at 120 dpi might have rendered the mice more sensitive to experimental manipulation, including ASO injection. Further studies are needed to understand this phenomenon. In the meantime, our results with active ASO 1 indicate that ASO-mediated PrP lowering can be both tolerated and efficacious even after advanced prion neuropathology is present, and they support further development of this modality.

Attempts to develop a drug for prion disease have illuminated several critical challenges along the road to translation. As we assess the promise of ASOs, we believe the data presented here offer reasons for cautious optimism. First, molecules effective against peripheral prion infection may lack sufficient CNS distribution to be effective against prion infections in the brain (52). This is not the case for ASOs: available data demonstrate that ASOs delivered by bolus intrathecal injection can achieve broad brain distribution and target engagement in humans and NHPs (23, 48), with pharmacokinetic and safety parameters that support a regimen of dosing every 1–4 months (29, 46). Positive clinical and safety data for intrathecally delivered ASOs for other neurological indications (29, 46, 48) will help to pave the way for a PrP-targeting ASO.

A second historical challenge in development of a prion therapeutic has been that treatments effective prophylactically or against early-stage prion infection may not be effective against late-stage prion infection (42, 45). For example, the small molecule IND24 quadrupled survival in prophylactically treated mice but was completely ineffective at 90 dpi (45). Here, we demonstrate that PrP-targeting ASOs are effective both prophylactically and against an established prion infection in the brain, at 120 dpi. Although the mice we treated did not exhibit frank clinical signs according to our definition, neuropathology is prominent by this stage (53), and some investigators have documented behavioral changes before this time point (54). Our work builds on previous studies in conditionally PrP-expressing mice, where eliminating or reducing PrP expression was beneficial even after pathology was well established and subtle behavioral changes were beginning to emerge (3, 4, 55). We extend these findings by showing that the benefit of lowering PrP can be achieved in a prion-infected WT animal as late as 120 dpi, with only partial PrP suppression, and by a therapeutic modality.

A final hurdle with other modalities has been that several small molecules with unknown mechanisms of action discovered in phenotypic screens have proven effective against experimental prion strains and yet lack activity against human prion strains (45, 56–58). Here, we have found that the benefit of PrP-targeting ASOs in vivo is driven by reduction of PrP mRNA and protein levels and not by aptameric binding of ASOs to PrP (35–37). Therefore, ASOs may prove effective across prion strains. As a whole, these data suggest that ASO-mediated suppression of PrP may offer broad efficacy against prion disease.

The benefit of ASOs in prophylactic treatment observed here may suggest a potential for PrP-lowering therapy to delay disease onset in presymptomatic individuals at risk for genetic prion disease. Predictive genetic testing makes it possible to identify individuals at >90% lifetime risk of prion disease (11), and while the variable age of onset poses a challenge for prevention trials (59), efforts are underway to enable informative clinical trials in this population (41, 51, 60). Meanwhile, the benefit of ASOs in late treatment may also suggest a potential for effective treatment in already-symptomatic individuals with prion disease. One challenge for studies in a symptomatic population is that prion disease is exceptionally rapid, with a median duration of only 5 months (61), and patients usually spend most of this time searching for a diagnosis (62, 63), meaning that most patients have advanced symptoms by the time they can be identified. Time to diagnosis will be a critical variable for reaching patients early enough. Real-time quaking-induced conversion (RT-QuIC) now offers a highly sensitive and specific diagnostic assay (64–67), but for this assay to reach its potential, we must strive to elevate the profile of prion disease in the differential diagnosis of rapidly progressive dementias.

Our work also provides a particularly clear demonstration of the potential for ASOs to effectively treat neurodegenerative disorders by lowering a target protein in the brain. ASOs designed to lower various CNS target proteins have already shown preclinical efficacy, but always either in transgenic mice (20–27) or in WT mice induced to develop a pathology but without frank disease (68). Unlike other neurodegenerative diseases, prion disease afflicts a wide variety of mammalian species, and transmission of prions to mice leads to a uniquely aggressive symptomatic course culminating in fatal disease. Thus, our studies demonstrate not only effective treatment of a model, but effective treatment of a rapidly fatal neurodegenerative disease in a WT organism. ASOs designed to lower a target protein in the brain, thus, have enormous potential in the treatment of neurodegeneration.

## Methods

### Study design and sites.

We sought to determine whether ASOs could sequence-specifically lower PrP in the mouse brain and extend survival in prion-infected mice. Scientists at Ionis Pharmaceuticals led the discovery of ASOs and characterization of their potency by reverse transcription PCR (RT-PCR) in both cells and animals. Experiments in prion-infected animals were conducted concurrently at 2 sites — NIH/NIAID/Rocky Mountain Laboratories and the Broad Institute of MIT and Harvard. NIH also characterized ASO potency effects on PrP by protein quantification and IHC.

ASO treatment studies were designed as controlled laboratory studies, with a primary endpoint of terminal prion disease requiring euthanasia. At NIH, animals were also monitored for clinical onset according to prespecified clinical neurological signs: progressive deterioration of ataxia, tremors, myoclonus, weight loss, somnolence, kyphosis, and poor grooming. At NIH, some animals were prespecified for intercurrent euthanasia to serve as time point–matched controls in biochemical and histological analyses, and they were excluded from analyses of prion disease onset and survival. Animals that died of nonprion causes were included in calculations of all-cause mortality but were excluded from analyses of prion disease symptom onset. At Broad Institute, animals were monitored once per week, increasing to every other day after 120 dpi, for the following signs: generalized tremor, ataxia, difficulty righting from a supine position, rigidity of the tail, stare or blank look, and hindlimb weakness; animals were weighed every other day after 120 dpi and were euthanized upon body condition score <2, body weight loss >20%, inability to reach food or water, severe respiratory distress, or severe neurological deficits.

### Animals.

Experiments used 6- to 12-week-old WT female mice, either C57BL/6N purchased from Taconic Biosciences Inc. or SWR/R mice, an outbred colony of primarily Swiss origins maintained at Rocky Mountain Laboratories for many generations (69). Both mouse strains harbor the *Prnp^a^* (MoPrP-A) haplotype (70), as found in the mouse reference genome. Figure 1, A, B, and D; Figure 2, A–C; Figure 3; Figure 4; and Supplemental Figures 2–6 display data from SWR/R mice. Figure 1, C–E, and Supplemental Figure 1 display data from C57BL/6N mice.

### ASO synthesis, screening, and lead identification.

Synthesis and purification of all chemically purified ASOs were performed as previously described (71). Approximately 500 ASOs were designed against the full mouse *Prnp^a^* gene. Electroporation of ASOs was carried out using the HT-200 BTX Electroporator with ElectroSquare Porator (ECM830) voltage source at 135 V in 96-well electroporation plates (BTX, 2 mm; Harvard Apparatus). ASOs were screened in HEPA1-6 cells (ATCC, 1830) at 7 μM. Cells were harvested at 24 hours after treatment for RNA extraction, and mouse *Prnp* mRNA was quantified by RT-PCR. The most potent ASOs were taken into a 4-point dose response in HEPA1-6 cells. Active ASO 1 and active ASO 2 were then characterized by screening in C57BL/6N mice by i.c. ventricular injection of 300 μg, with tissue collection at 2 weeks after dose for *Prnp* mRNA reduction (Supplemental Figure 1A). After synthesis, ASOs were aseptically diluted to 100 mg/ml in PBS and frozen at –20°C.

### Stereotactic i.c.v. injection of ASO or PBS at the NIH.

After thawing, the actual concentrations of each ASO were determined by spectroscopy at an absorbance of 260 nm. Based on absorbance calculated concentration values, each ASO was further diluted to 66.7 μg/μl in PBS, and 4.5 μl was injected i.c.v. into each mouse as described below. For buffer-only control mice, 4.5 μl of PBS was injected i.c.v. Aliquots of the ASO solutions were stored at –20°C for repeated ASO treatments, and a fresh aliquot was thawed and used for injections at each of the designated time points.

Two- to 3-month-old female mice were anesthetized with isoflurane and prepared for surgery by shaving the hair from the dorsal surface of the skull and applying chlorhexidine-based surgical scrub (BD Biosciences) to the area. Mice were then positioned on a stereotaxic frame (David-Kopf Instruments) and maintained on isoflurane anesthesia. Using aseptic technique, a 1-cm midline incision was made in the skin over the dorsal surface of the skull, and the skull was exposed to allow positioning of the drill over the bregma point of reference. Coordinates used from bregma were 0.0 mm anterioposterior, 0.8 mm lateral (right), and 2.5 mm ventral (down) to skull surface. These coordinates were selected to target the center of the lateral ventricle. A small hole was drilled in the surface of the skull prior to placement of the 32-gauge delivery needle (World Precision Instruments). A total volume of 4.5 μl containing 300 μg of ASO in PBS or PBS alone was injected into the ventricle at a rate of 1 μl/sec using an UltraMicroPump III with Micro4 pump controller (World Precision Instruments). The needle was kept in place for 1 minute following injection to minimize reflux. The skin incision was closed with suture. Mice were recovered in heated cages after surgery and received a single s.c. injection of 0.2 mg/kg buprenorphine (Par Pharmaceuticals) for postoperative pain management. Patency of the needles was verified prior to and after injections.

### Stereotactic i.c.v. injection of ASO or PBS at Ionis Pharmaceuticals and the Broad Institute.

Animals were induced with 3% isoflurane and maintained on 3% isoflurane for a surgical plane of anesthesia throughout the procedure. Mouse heads were shaved and swabbed with betadine, and animals received prophylactic meloxicam for pain relief. Animals were placed in a stereotaxic apparatus (ASI Instruments, SAS-4100) with the 18° ear bars in the ear canals and the incisors in the tooth bar of the mouse adapter, adjusted to –8 mm so that the bregma and lambda landmarks on the skull were level. After making an approximately 1-cm incision in the scalp, s.c. tissue and periosteum were scrubbed from the skull with sterile cotton-tipped applicators to reveal the bregma. Hamilton syringes (VWR 60376-172) fitted with 22-gauge Huber point removable needles (VWR 82010-236) were filled with 10 μl of saline with or without ASO (diluted from 100 mg/ml in DPBS, Thermo Fisher Scientific, 14190). The needle was positioned over bregma and then moved to coordinates 0.3 mm anterior, 1.0 mm right, and 3.0 mm down after the bevel of the needle disappears through the skull. Saline (10 μl) was injected gradually by hand over approximately 10 seconds. After 3 minutes, the needle was backed out of the skull while applying downward pressure on the animal’s skull with a sterile cotton-tipped applicator. The incision was sutured with 1 horizontal mattress stitch using 5-O Ethilon suture (Ethicon, 661H). Animals were allowed to recover from the anesthesia in their home cage. For the data in Figure 2, D–E, the first round of injections were performed at Ionis Pharmaceuticals at –14 dpi, and animals were then shipped to the Broad Institute for prion inoculation and a subsequent second round of i.c.v. injections at 76 dpi (90 days after the first injections).

### I.c. prion inoculations at the NIH.

Per the time course shown in Figures 2 and 3, 8 to 12-week-old female mice were injected i.c. with 25 μl of 1% brain homogenate in PBS prepared aseptically from a pool of 10 RML prion–infected mouse brains excised at terminal stage of rodent-adapted scrapie (1 × 10^8.3^ ID_50_/gram of brain). A 10% brain homogenate was aseptically prepared in 0.32 M sucrose by douncing the pool of excised brains 10 times each, first with the loose pestle and then with the tight pestle (Wheaton glass); sonicated for 2 pulses of 1 minute each (held for 30 seconds on ice in between pulses) in a cuphorn sonicator at maximum setting, followed by centrifugation at 1,500 *g* for 5 minutes; and resulting supernatant was used for inoculations. Inocula was aliquoted and stored at –80°C, and a fresh aliquot was used for each set of inoculations after it was rapidly thawed at 37°C, sonicated in a cuphorn sonicator at maximum setting for 2 sonication pulses as before, and dilution was done in PBS (Amresco). This was made and held at room temperature just prior to inoculations. Mice were restrained during i.c. injections by anesthesia using saturated isofluorane vapors in a bell jar until the mice were unconscious.

### I.c. prion inoculations at the Broad Institute.

Animals were 7–10 weeks old at the time of inoculation so that skulls were cartilaginous enough to allow manual i.c. inoculation. Each animal received 30 μl of a 1% RML prion brain homogenate, extruded through successively smaller-gauge needles, and irradiated at approximately 7.0 kGy to kill opportunistic pathogens prior to injection (72). Brain homogenate was loaded into a 300-μl BD SafetyGlide Insulin 31G syringe with a 6 mm needle (BD Biosciences, 328449). Mice were induced and maintained on a surgical plane of anesthesia with 3% isoflurane. Mouse heads were wiped with betadine. The needle was manually inserted through the skull, the plunger was depressed, and, after 3 seconds, it was removed. Animals were allowed to recover in their home cages.

### RT-PCR.

Cultured cells were lysed in 300 μl of RLT buffer (Qiagen) containing 1% (v/v) 2-mercaptoethanol (BME, MilliporeSigma). “For RNA extraction, the following were dissected: (a) a 2-mm coronal section of the cortex at 1 mm posterior to the injection site, (b) the hippocampus, and (c) a 2-mm coronal section of the thoracic spinal cord. Tissues were homogenized in 500 μl of RLT buffer containing 1% (v/v) BME. RNA was isolated from 20 μl of lysate with an RNeasy 96 Kit (Qiagen) that included in-column DNA digestion with 50 U of DNase I (Invitrogen). RT-PCR was performed using StepOne Realtime PCR system (Applied Biosystems), as described previously (50). The sequences of primers and probes are provided in Supplemental Table 1. PCR results were normalized by housekeeping gene cyclophilinA/Ppia and further normalized to the level in PBS-treated mice or untreated cells.

### PrP immunoblot analysis of brain tissues for total PrP and PK-resistant PrP.

After euthanasia and dissection, half of a sagittally divided mouse brain was flash frozen in liquid nitrogen and stored at –80°C; the other half was put into neutralized 10% formalin and used for immunohistochemical analysis. For immunoblot analysis, brains were thawed on ice. While thawing, the mass of each brain sample was determined; then, cold sterile phosphate buffered saline was added in order to make a 20% (weight/volume [w/v]) brain homogenate in a 2-ml polypropylene microcentrifuge tube containing 0.6 mg of 1 mm zircon beads (BioSpec). The tubes were then shaken in a bead beater (Bead Mill 24, Thermo Fisher Scientific) at maximum setting for 1 minute and placed on ice. Immediately afterward, the samples were centrifuged for 2 minutes at 2,000 *g*. The supernatant was aliquoted and returned to ice if immunobloted immediately or frozen at –80°C. To determine amount of total PrP, the brain homogenates were diluted to 1% in 0.04 ml total volume in 1× Sabu (0.0625 M Tris-Cl [pH 6.8], 0.003 M EDTA, 5% glycerol, 5% SDS, 4% β-mercaptoethanol, 0.02% bromophenol blue [MilliporeSigma]), vortexed well, and boiled for 5 minutes. Of the resulting sample, 10 μl was loaded onto 10% Bis-Tris NuPAGE polyacrylamide gels (Invitrogen) for electrophoresis. For analysis of PK-resistant PrP, 5 μl of 20% brain homogenate was diluted into 0.1 M Tris-Cl (pH 8.5), 0.15 M NaCl, 0.001 M CaCl_2_, and 50 μg/ml PK (Calbiochem) and incubated at 37°C for 1 hour. Then, 1 μl of 0.1 M Pefabloc (Fluka) was added; samples were vortexed and incubated on ice for 5 minutes; 55 μl of 2× Sabu was added, vortexed, and boiled for 5 minutes; and 10 μl of each sample was loaded onto gels as above. All gels were blotted onto PVDF membranes (MilliporeSigma) in Towbin buffer (0.025 M Tris base, 0.192 M glycine, 20% methanol) using a semidry blotter (Biometra) per manufacturer’s recommendations. Membranes were placed into a 50-ml polypropylene conical tube with the protein side facing inward and blocked using 5% Blotting-Grade Blocker (Bio-Rad) in TBST buffer (0.01 M Tris base, 0.15 M NaCl, 0.05% Tween 20) for 1 hour with continuous gentle rolling rotation. Blocking solution was removed and replaced with 10 ml mAb 6D11 (Santa Cruz Biotechnology Inc.) diluted 1:5,000 in blocking solution and incubated for 1 hour at room temperature. The membranes were washed in the same tube for 5 minutes, 4 times sequentially using 40 ml TBST dispensed into the tube. Next, the membranes were incubated/rotated for 1 hour in 10 ml goat anti–mouse alkaline phosphatase (Thermo Fisher Scientific, A16069) diluted 1:10,000 in blocking solution. After this incubation, membranes were washed 5 times for 5 minutes sequentially as above. Membranes were placed protein-side down into a square petri dish with 1.25 ml of AttoPhos solution (Promega) for 5 minutes and then air-dried overnight while hanging from a clip. Membranes were placed into a nonfluorescing plastic sheet protector and scanned with a Typhoon FLA 9500 (GE Healthcare) fluorescence imager. Resulting images were quantified using ImageQuant software (GE Healthcare).

### IHC and histology.

Brains were removed and cut in half in the sagittal plane, and one half of each brain was placed in 10% neutral buffered formalin for 3–5 days. Tissues were then processed by dehydration and embedding in paraffin. Sections were cut using a standard Leica microtome, placed on positively charged glass slides, and air-dried overnight at room temperature. On the following day, slides were heated in an oven at 60°C for 20–30 minutes.

For PrP, glial fibrillary acidic protein (GFAP), and ASO immunohistochemical staining, all deparaffinization, antigen retrieval, and staining were performed using the Ventana automated Discovery XT stainer. PrP^Sc^ staining requires aggressive antigen retrieval using high temperatures to expose the epitopes. Antigen retrieval for PrP^Sc^ staining was performed by incubation in CC1 buffer (Ventana) containing Tris-borate-EDTA (pH 8.0; MilliporeSigma), for 100 minutes at 95°C. Immunohistochemical staining for PrP was done using human anti–PrP monoclonal D13 antibody (73) in tissue culture fluid at a dilution of 1:100 for 2 hours at 37°C. The secondary antibody was biotinylated goat anti–human IgG at a 1:250 dilution (Jackson ImmunoResearch), and streptavidin-biotin peroxidase was used with DAB as chromogen (DAB Map kit; Ventana Medical Systems). For GFAP staining of astrocytes, antigen retrieval was done by using the Discovery XT system with the mild CC1 protocol (cell conditioning buffer containing Tris-borate-EDTA [pH 8.0], with incubation for 12 minutes at 100°C). The anti-GFAP antibody (74) was used at a dilution of 1:3,500 in antibody dilution buffer (Ventana, ADB250), applied for 16 minutes at 37°C. The secondary antibody was biotinylated goat anti–rabbit IgG (Biogenex Ready-to-use Super Sensitive Rabbit Link, HK3369R), applied for 16 minutes at 37°C. Staining was completed by using a RedMap detection kit. To stain ASO distribution in tissues, slides were pretreated with PK (DAKO Ready-to-Use) for 4 minutes at 37°C. The previously described (22) anti-ASO antibody (New Zealand rabbit polyclonal serum #6651, Ionis Pharmaceuticals) was applied at a 1:20,000 dilution in antibody dilution buffer (Ventana ADB250) for 60 minutes at 37°C. The secondary antibody was biotinylated goat anti–rabbit IgG (Biogenex, HK3369R) applied for 32 minutes at 37°C. The DAB Map Kit was applied as described above. For all IHC slides, hematoxylin was used as the counterstain and antibody diluent alone was used as a negative control. H&E staining was performed according to the manufacturer’s (Shandon) instructions: hematoxylin incubation of 12 minutes and eosin incubation of 4 minutes. Sections stained with D13, anti-GFAP, anti-ASO, and H&E were scanned with an Aperio ScanScope XT (Aperio Technologies Inc.), and they were analyzed and photographed using Aperio Imagescope software. Sections that were IHC stained for ASO and PrP were scanned with an Aperio ScanScope XT (Aperio Technologies Inc.) and quantified using the ImageScope positive pixel count algorithm (version 9.1). For each brain, a 5-μm–thick median sagittal section representing approximately 55 mm^2^ was evaluated. The pixel-counting algorithm interpreted the darkness or lightness of each pixel intensity and divided the data into categories based on intensity. The darkest staining possible (black) was given a score of 0, and the lightest staining (white) pixel score was 255. Hues of brown produced by DAB IHC detection systems used for the ASO and PrP staining score positive using this algorithm. For both ASO and PrP pixel scoring, all positive pixels (scores of 0–220) and negative pixels (scores of 221–255) were counted. To calculate the percentage of positive pixels in each tissue section, the following formula was used: positive pixels/total pixels × 100.

### Statistics.

For survival experiments, disease onset and survival endpoints were plotted as Kaplan-Meier survival curves. Differences between groups were considered visually obvious; statistical tests were not used. For ASO-mediated RNA reduction quantification, days from onset to terminal disease, and quantification of immunohistochemical markers, 95% CIs were calculated as ±1.96 times the SEM. Data were analyzed using GraphPad Prism (NIH) or R 3.5.1 (Broad Institute). Data and source codes sufficient to reproduce the figures herein are provided in a GitHub repository (https://github.com/ericminikel/aso_survival, under release 1.0; https://github.com/ericminikel/aso_survival/releases/tag/v1.0).

### Study approval.

All experimental procedures involving animals were approved by IACUCs (Ionis IACUC protocol P-0273, Broad Institute IACUC protocol 0162-05-17, and NIH IACUC protocol 2015-061) and were performed in accordance with the NIH *Guide for the Care and Use of Laboratory Animals* (National Academies Press, 2011).

## Author contributions

SMV, BC, HTZ, HK, GJR, EVM, and JBC conceived and designed the study. GJR, HTZ, BR, LDR, KW, AK, AGR, SG, JL, TC, EVM, CM, DEC, LLL, and SMV performed the experiments. BC, SMV, SLS, JS, RO, TC, HK, and EES supervised the research. SMV, BC, GJR, EVM, HTZ, and BR wrote the paper. All authors reviewed and edited the paper.

## Supplementary Material

Supplemental data

## Figures and Tables

**Table 1:**
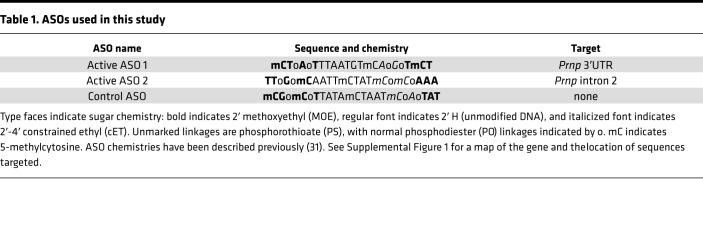
ASOs used in this study

## Version 1 07/30/2019

In-Press Preview

## Version 2 08/22/2019

Electronic publication
