# Supplementary material for: Antisense oligonucleotides extend survival of prion-infected mice
Source: JCI Insight. 2019 Aug 22;4(16):e131175. doi: 10.1172/jci.insight.131175 (PMC6777807; doi:10.1172/jci.insight.131175)
Supplement: Supplemental data [file jciinsight-4-131175-s170.pdf]

SUPPLEMENTAL DATA

Antisense oligonucleotides extend survival of prion-infected mice

Gregory J Raymond[1], Hien Tran Zhao[2], Brent Race[1], Lynne D Raymond[1], Katie Williams[1], Eric Swayze[2], Samantha Graffam[3], Jason Le[3], Tyler Caron[3], Jacquelyn Stathopoulos[3], Rhonda O'Keefe[3], Lori L Lubke[1], Andrew G. Reidenbach[3], Allison Kraus[1], Stuart L Schreiber[3], Curt Mazur[2], Deborah E Cabin[4], Jeffrey B Carroll[5], Eric Vallabh Minikel[1,3,6,7], Holly Kordasiewicz[2], Byron Caughey[1,†], Sonia M Vallabh[1,3,6,7,†]

Supplementary Figures

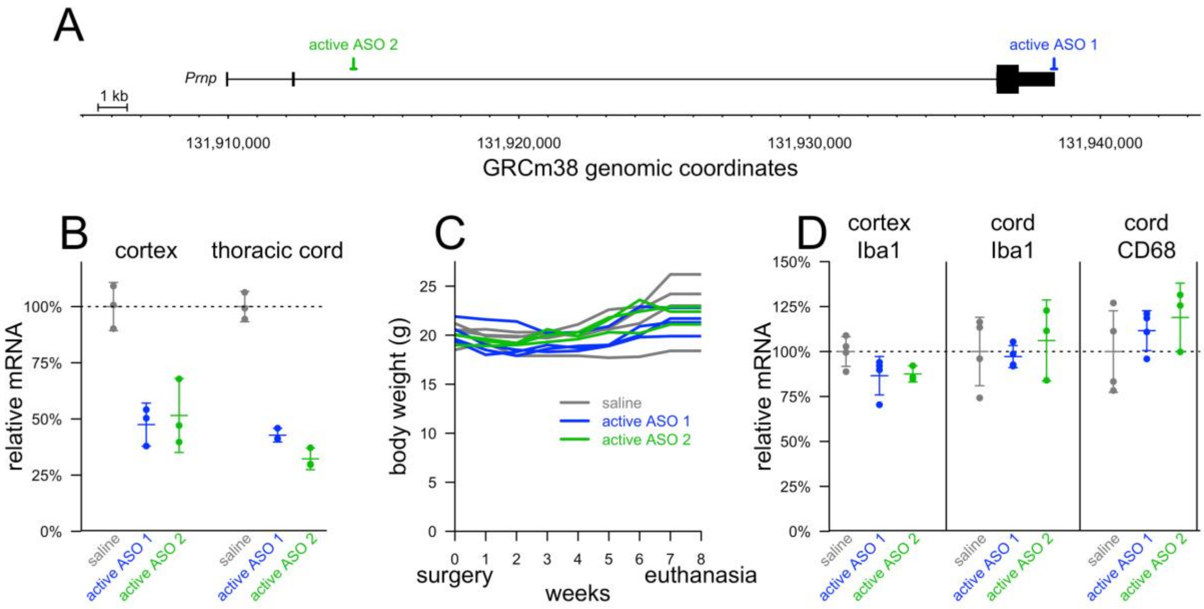

Figure S1. Screening of PrP-lowering ASOs. A) *Genomic location of sequences targeted by active ASOs 1 and 2. GRCm38 reference sequence, chromosome 2. Gene structure represented by line (introns), medium bar (5' and 3'UTR), and thick bar (open reading frame).* ***B)*** *C57BL/6N animals (N=3 per group) were given a single 300µg ICV bolus dose of the indicated ASOs and ipsilateral PrP mRNA was quantified by qPCR 2 weeks later.* ***C-D)*** *C57BL/6N animals (N=4 per group, of which one active ASO 2 animal died of surgical complications) were given a single 700 µg ICV bolus dose. Body weights (C) were monitored for 8 weeks, and then animals were sacrificed for qPCR analysis (D) of inflammatory markers. In B and D, dots represent individual animals, and bars represent mean and 95% confidence intervals.*

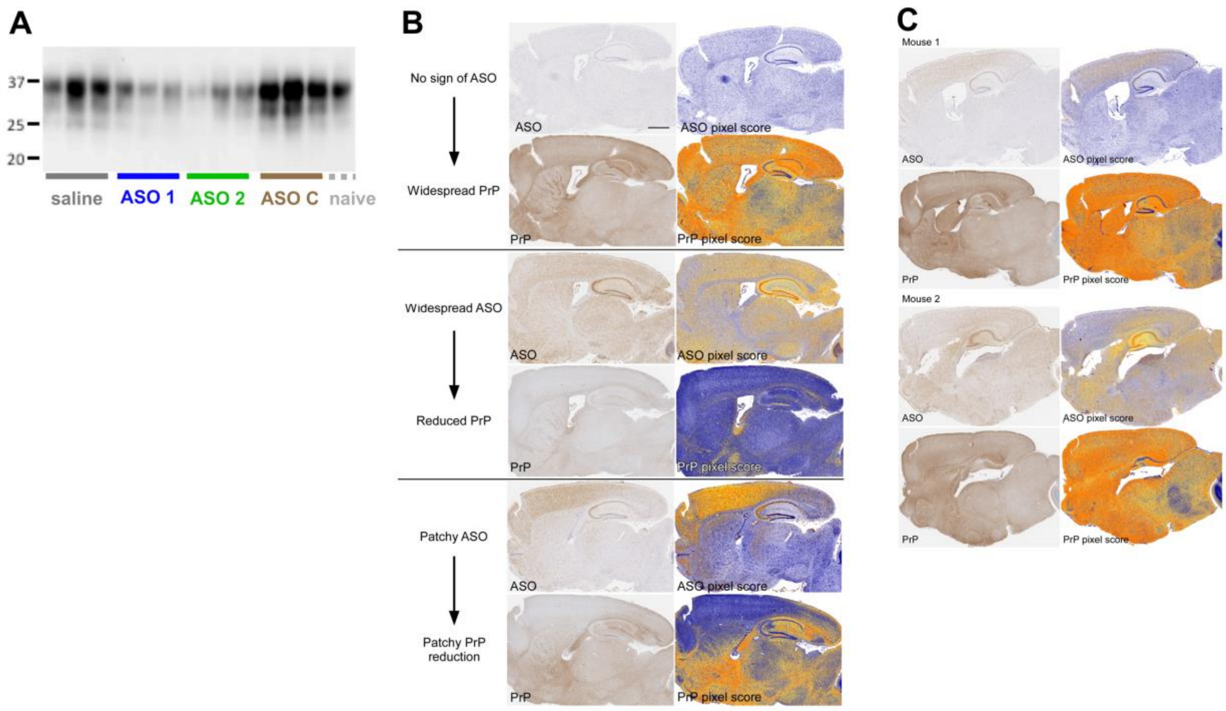

Figure S2. Additional data regarding potency of PrP-lowering ASOs. *A) Mice received two 300µg ICV ASO doses at 60-day intervals and PrP in contralateral hemispheres was quantified 60 days after the second dose. Primary antibody used for PrP detection was 6D11. The migration of molecular mass standards in kilodaltons is shown on the right of the blot. B) Inverse distributions of anti-PrP ASOs and PrP 18 days after intracerebral injection of 500 µg of the ASO. Immunohistochemical (IHC) staining for ASO and PrP as designated on midline sagittal sections of mouse brains (left column) with false color heat maps based on pixel scoring (blue = low, orange = high). Due to inconsistent drug delivery with freehand dosing, different outcomes were observed across different animals, and three scenarios are shown here: low ASO 1 retention in brain with strong PrP staining (top); widespread ASO 1 with low PrP (center); patchy ASO 2 distribution with inverse PrP distribution (bottom). Representative images chosen from a total of 9 animals (6 active ASO 1 and 3 active ASO 2) analyzed. C) Equivalent to Figure S2B but for control ASO. Here, no inverse relationship between ASO and PrP distribution is observed. Representative images chosen from a total of 3 animals analyzed.*

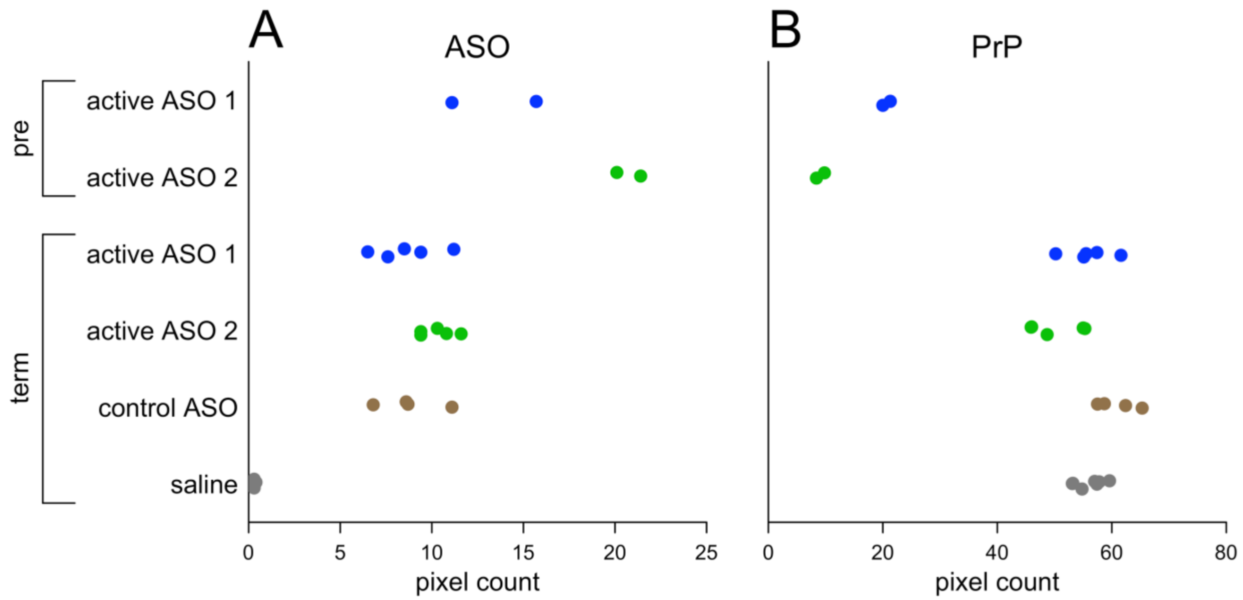

Figure S3. Quantification of PrP and ASO immunohistochemical staining in additional prophylactically treated animals. *Pixel counts of ASO (left) and PrP (right) staining in whole sagittal slices of brain from mice treated as designated at their terminal clinical endpoint (term) or, in 2 cases each of ASO 1- and 2-treated mice, in the preclinical (pre) phase when collected within several dpi of the terminal endpoint for the control mice. Data points represent individual mice (biological replicates).*

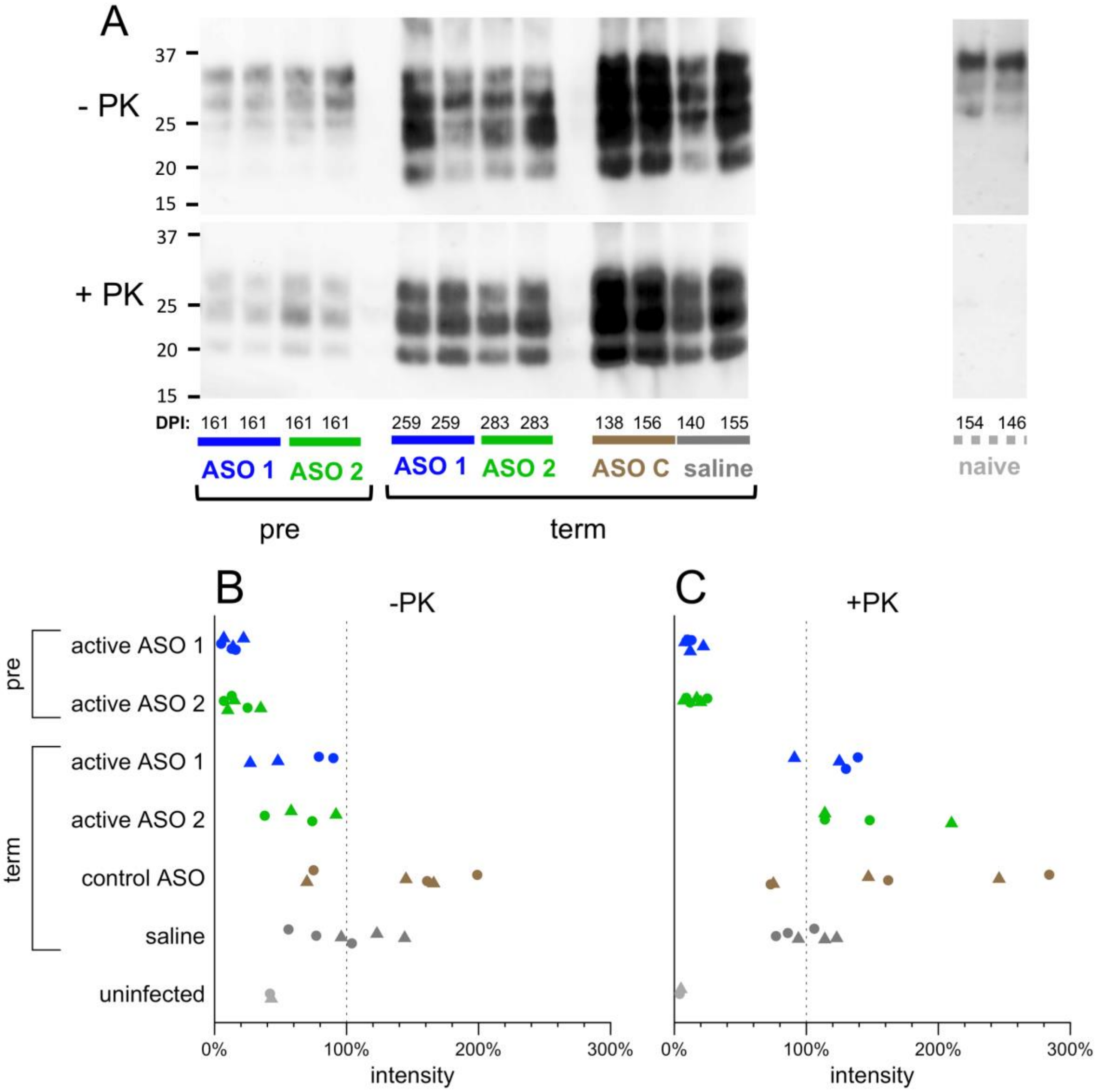

Figure S4. Immunoblotting analysis of total (-PK) or PK-resistant PrP (+PK) in brain tissue from prion-infected mice given ASO or control treatments prophylactically. A) *Western blots. The ASO saline treatments, the days post scrapie infection (DPI) that the animals were euthanized, and clinical status of the mice at death are designated as "pre" for preclinical, and "term" for the terminal clinical endpoint. "Naïve" (uninfected) mice were neither treated with ASOs nor inoculated with prions, and were run on separate blots shown at far right. The migration of molecular mass standards in kilodaltons is shown on the left of each blot.* ***B-C)*** *Pixel count-based quantification of Western blots without (B) and with (C) proteinase K (PK) digestion, normalized to the mean value for saline-treated animals as 100%. Within each row, differently shaped symbols represent different mice, and the multiple data points for each mouse represent technical replicates from different Western blots.*

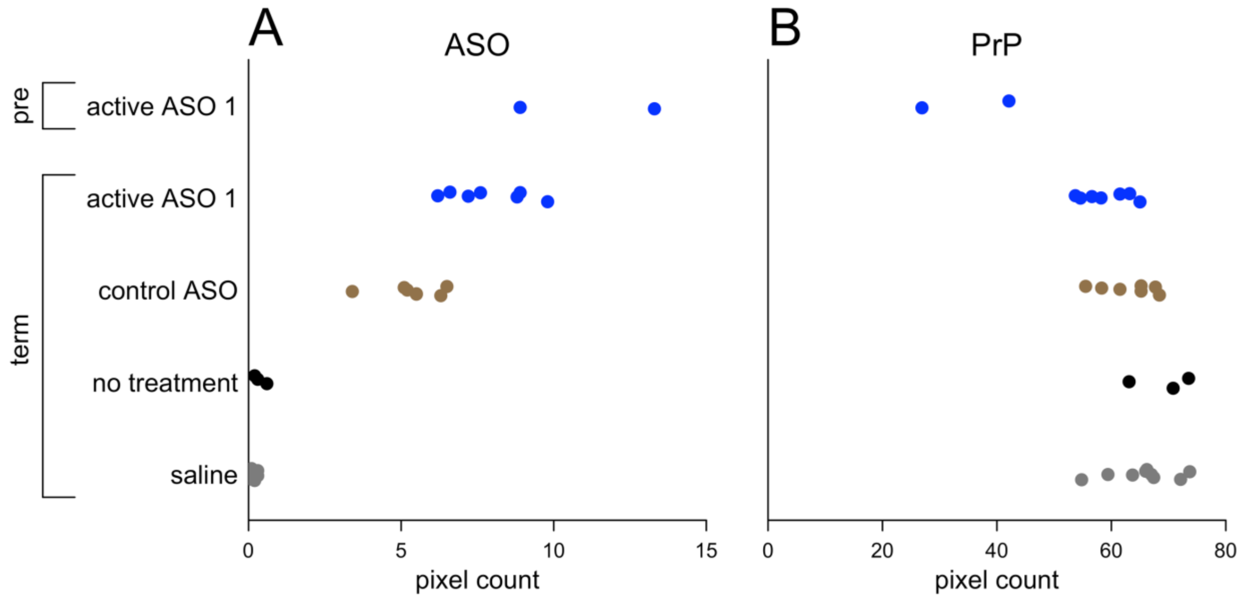

Figure S5. Quantification of PrP and ASO immunohistochemical staining in additional 120 dpi-treated animals. *Pixel counts of ASO (left) and PrP (right) staining in whole sagittal slices of brain from mice treated designated at their terminal clinical endpoint (term) or, for two ASO 1 mouse, in the preclinical (pre) phase when collected within several dpi of the terminal endpoint of the control mice. Data points represent individual mice (biological replicates).*

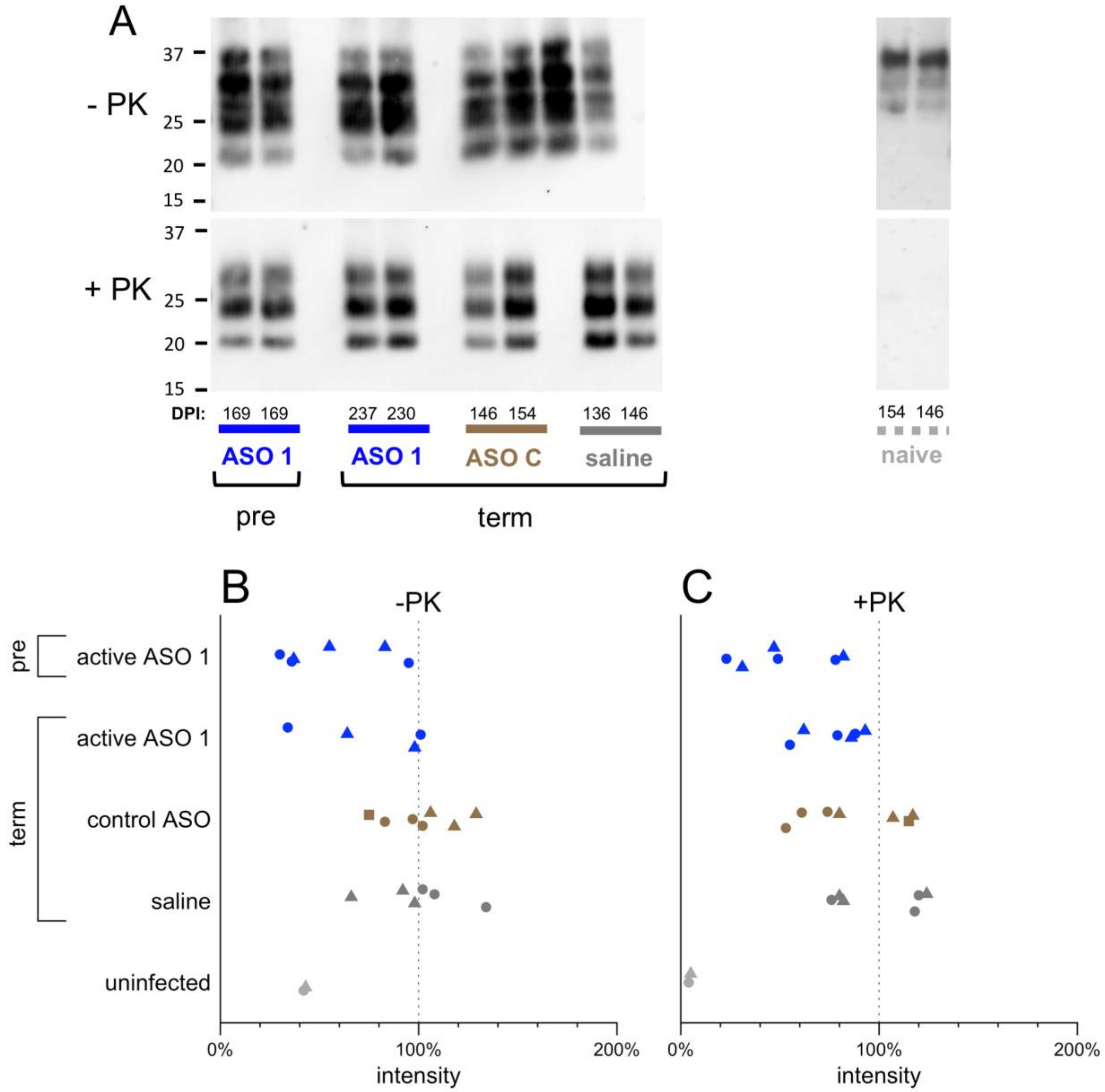

Figure S6. Immunoblotting analysis of total (-PK) or PK-resistant PrP (+PK) in brain tissue from prion-infected mice given ASO or control treatments at 120 dpi. A) *Western blots. The ASO or saline treatments, the days post scrapie infection (DPI) that the animals were euthanized, and clinical status of the mice at death are designated as "pre" for preclinical, and "term" for the terminal clinical endpoint. The same two Naïve uninfected mice from Figure S4 are reproduced here for comparison, with separate blots again shown at far right. The migration of molecular mass standards in kilodaltons is shown on the left of each blot.* ***B-C)*** *Pixel count-based quantification of Western blots without (B) and with (C) proteinase K (PK) digestion, normalized to the mean value for saline-treated animals as 100%. Within each row, differently shaped symbols represent different mice, and the multiple data points for each mouse represent technical replicates from different Western blots.*

Supplementary Tables

Table S1. Primers and probes used for RT-PCR. *Cyclophilin was used for normalization (see Methods).*

gene	primer	sequence
Prnp	Forward	TCAGTCATCATGGCGAACCTT
	Reverse	AGGCCGACATCAGTCCACAT
	Probe	CTACTGGCTGCTGGCCCTCTTTGTGACX
**cyclophilin**	Forward	TCGCCGCTTGCTGCA
	Reverse	ATCGGCCGTGATGTCGA
	Probe	CCATGGTCAACCCCACCGTGTTCX
***Aif1* (Iba1)**	Forward	TGGTCCCCCAGCCAAGA
	Reverse	CCCACCGTGTGACATCCA
	Probe	5'-Fam-AGCTATCTCCGAGCTGCCCTGATTGGX-Tamra-3'
Cd68	Forward	TGGCGGTGGAATACAATGTG
	Reverse	GATGAATTCTGCGCCATGAA
	Probe	CCTTCCCACAGGCAGCACAGTGGX

Table S2. Details on all mice. *The all-cause mortality column indicates whether the animal is counted as a death (1) or censored (0) for the all-cause mortality curves shown in figures 2C, 2E, and 3C. *One animal died without developing clinical signs of prion disease, but was positive for prion pathology by immunohistochemistry.*

experiment	treatment	dpi	cause of death	all-cause mortality
NIH prophylactic	no treatment	92	intercurrent	1
NIH prophylactic	no treatment	136	prion disease	1
NIH prophylactic	no treatment	139	prion disease	1
NIH prophylactic	no treatment	140	prion disease	1
NIH prophylactic	no treatment	146	prion disease	1
NIH prophylactic	no treatment	146	prion disease	1
NIH prophylactic	no treatment	152	prion disease	1
NIH prophylactic	no treatment	157	prion disease	1
NIH prophylactic	saline	97	intercurrent	1
NIH prophylactic	saline	118	intercurrent	1
NIH prophylactic	saline	140	prion disease	1
NIH prophylactic	saline	140	prion disease	1
NIH prophylactic	saline	143	prion disease	1
NIH prophylactic	saline	155	prion disease	1
NIH prophylactic	saline	155	prion disease	1
NIH prophylactic	saline	156	prion disease	1
NIH prophylactic	saline	156	prion disease	1
NIH prophylactic	control ASO	70	intercurrent	1
NIH prophylactic	control ASO	120	sudden	1

experiment	treatment	dpi	cause of death	all-cause mortality
NIH prophylactic	control ASO	120	sudden	1
NIH prophylactic	control ASO	120	sudden	1
NIH prophylactic	control ASO	121	sudden	1
NIH prophylactic	control ASO	138	prion disease	1
NIH prophylactic	control ASO	149	prion disease	1
NIH prophylactic	control ASO	156	prion disease	1
NIH prophylactic	control ASO	156	prion disease	1
NIH prophylactic	active ASO 1	144	intercurrent	1
NIH prophylactic	active ASO 1	161	sacrificed for histology	0
NIH prophylactic	active ASO 1	161	sacrificed for histology	0
NIH prophylactic	active ASO 1	227	unknown*	1
NIH prophylactic	active ASO 1	259	prion disease	1
NIH prophylactic	active ASO 1	259	prion disease	1
NIH prophylactic	active ASO 1	267	prion disease	1
NIH prophylactic	active ASO 1	267	prion disease	1
NIH prophylactic	active ASO 1	528	never developed disease	0
NIH prophylactic	active ASO 2	113	intercurrent	1
NIH prophylactic	active ASO 2	161	sacrificed for histology	0
NIH prophylactic	active ASO 2	161	sacrificed for histology	0
NIH prophylactic	active ASO 2	241	intercurrent	1
NIH prophylactic	active ASO 2	283	prion disease	1
NIH prophylactic	active ASO 2	283	prion disease	1
NIH prophylactic	active ASO 2	283	prion disease	1
NIH prophylactic	active ASO 2	286	prion disease	1
NIH prophylactic	active ASO 2	290	prion disease	1
NIH 120 dpi	saline	136	prion disease	1
NIH 120 dpi	saline	146	prion disease	1
NIH 120 dpi	saline	152	prion disease	1
NIH 120 dpi	saline	154	prion disease	1
NIH 120 dpi	saline	157	prion disease	1
NIH 120 dpi	saline	157	prion disease	1
NIH 120 dpi	saline	157	prion disease	1
NIH 120 dpi	saline	163	prion disease	1
NIH 120 dpi	saline	163	prion disease	1
NIH 120 dpi	control ASO	128	sudden	1
NIH 120 dpi	control ASO	129	sudden	1
NIH 120 dpi	control ASO	146	prion disease	1
NIH 120 dpi	control ASO	152	prion disease	1
NIH 120 dpi	control ASO	154	prion disease	1

experiment	treatment	dpi	cause of death	all-cause mortality
NIH 120 dpi	control ASO	154	prion disease	1
NIH 120 dpi	control ASO	154	prion disease	1
NIH 120 dpi	control ASO	154	prion disease	1
NIH 120 dpi	control ASO	163	prion disease	1
NIH 120 dpi	active ASO 1	169	sacrificed for histology	0
NIH 120 dpi	active ASO 1	169	sacrificed for histology	0
NIH 120 dpi	active ASO 1	230	prion disease	1
NIH 120 dpi	active ASO 1	237	prion disease	1
NIH 120 dpi	active ASO 1	244	prion disease	1
NIH 120 dpi	active ASO 1	244	prion disease	1
NIH 120 dpi	active ASO 1	248	prion disease	1
NIH 120 dpi	active ASO 1	248	prion disease	1
NIH 120 dpi	active ASO 1	257	prion disease	1
NIH 120 dpi	active ASO 2	129	sudden	1
NIH 120 dpi	active ASO 2	129	sudden	1
NIH 120 dpi	active ASO 2	129	sudden	1
NIH 120 dpi	active ASO 2	129	sudden	1
NIH 120 dpi	active ASO 2	130	sudden	1
NIH 120 dpi	active ASO 2	130	sudden	1
NIH 120 dpi	active ASO 2	130	sudden	1
NIH 120 dpi	active ASO 2	130	sudden	1
NIH 120 dpi	active ASO 2	130	sudden	1
Broad prophylactic	no treatment	1	intercurrent	0
Broad prophylactic	no treatment	1	intercurrent	0
Broad prophylactic	no treatment	139	prion disease	1
Broad prophylactic	no treatment	141	prion disease	1
Broad prophylactic	no treatment	155	prion disease	1
Broad prophylactic	no treatment	155	prion disease	1
Broad prophylactic	no treatment	157	prion disease	1
Broad prophylactic	no treatment	157	prion disease	1
Broad prophylactic	no treatment	157	prion disease	1
Broad prophylactic	no treatment	159	prion disease	1
Broad prophylactic	no treatment	160	prion disease	1
Broad prophylactic	no treatment	167	prion disease	1
Broad prophylactic	no treatment	169	prion disease	1
Broad prophylactic	no treatment	170	prion disease	1
Broad prophylactic	no treatment	171	prion disease	1
Broad prophylactic	no treatment	172	prion disease	1
Broad prophylactic	no treatment	172	prion disease	1

experiment	treatment	dpi	cause of death	all-cause mortality
Broad prophylactic	no treatment	183	prion disease	1
Broad prophylactic	no treatment	183	prion disease	1
Broad prophylactic	no treatment	206	prion disease	1
Broad prophylactic	saline	157	prion disease	1
Broad prophylactic	saline	168	prion disease	1
Broad prophylactic	saline	168	prion disease	1
Broad prophylactic	saline	169	prion disease	1
Broad prophylactic	saline	169	prion disease	1
Broad prophylactic	saline	170	prion disease	1
Broad prophylactic	saline	170	prion disease	1
Broad prophylactic	saline	170	prion disease	1
Broad prophylactic	saline	171	prion disease	1
Broad prophylactic	saline	171	prion disease	1
Broad prophylactic	saline	172	prion disease	1
Broad prophylactic	saline	172	prion disease	1
Broad prophylactic	control ASO	6	intercurrent	1
Broad prophylactic	control ASO	165	prion disease	1
Broad prophylactic	control ASO	165	prion disease	1
Broad prophylactic	control ASO	165	prion disease	1
Broad prophylactic	control ASO	167	prion disease	1
Broad prophylactic	control ASO	170	prion disease	1
Broad prophylactic	control ASO	170	prion disease	1
Broad prophylactic	control ASO	172	prion disease	1
Broad prophylactic	control ASO	172	prion disease	1
Broad prophylactic	control ASO	178	prion disease	1
Broad prophylactic	control ASO	190	prion disease	1
Broad prophylactic	control ASO	315	never developed disease	0
Broad prophylactic	active ASO 1	0	intercurrent	0
Broad prophylactic	active ASO 1	252	prion disease	1
Broad prophylactic	active ASO 1	263	prion disease	1
Broad prophylactic	active ASO 1	264	prion disease	1
Broad prophylactic	active ASO 1	270	prion disease	1
Broad prophylactic	active ASO 1	270	prion disease	1
Broad prophylactic	active ASO 1	277	prion disease	1
Broad prophylactic	active ASO 1	277	prion disease	1
Broad prophylactic	active ASO 1	277	prion disease	1
Broad prophylactic	active ASO 1	277	prion disease	1
Broad prophylactic	active ASO 1	281	prion disease	1
Broad prophylactic	active ASO 1	287	prion disease	1

experiment	treatment	dpi	cause of death	all-cause mortality
Broad prophylactic	active ASO 2	1	intercurrent	0
Broad prophylactic	active ASO 2	287	prion disease	1
Broad prophylactic	active ASO 2	294	prion disease	1
Broad prophylactic	active ASO 2	295	prion disease	1
Broad prophylactic	active ASO 2	299	prion disease	1
Broad prophylactic	active ASO 2	299	prion disease	1
Broad prophylactic	active ASO 2	301	prion disease	1
Broad prophylactic	active ASO 2	301	prion disease	1
Broad prophylactic	active ASO 2	301	prion disease	1
Broad prophylactic	active ASO 2	301	prion disease	1
Broad prophylactic	active ASO 2	303	prion disease	1
Broad prophylactic	active ASO 2	306	prion disease	1
